# High Capacity Prismatic Type Layered Electrode with Anionic Redox Activity as an Efficient Cathode Material and PVdF/SiO_2_ Composite Membrane for a Sodium Ion Battery

**DOI:** 10.3390/polym12030662

**Published:** 2020-03-16

**Authors:** Arjunan Ponnaiah, Subadevi Rengapillai, Diwakar Karuppiah, Sivakumar Marimuthu, Wei-Ren Liu, Chia-Hung Huang

**Affiliations:** 1 #120, Energy Materials Lab, Department of Physics, Science Block, Alagappa University, Karaikudi 630 003, Tamil Nadu, India; nano.arjun@gmail.com (A.P.); selfindicator@gmail.com (D.K.); 2 Department of Chemical Engineering, R&D Center for Membrane Technology, Research Center for Circular Economy, Chung-Yuan Christian University, Chung-Li, 32023, Taiwan; wrliu@cycu.edu.tw; 3 Metal Industries Research and Development Centre, Kaohsiung 81160, Taiwan; chiahung@mail.mirdc.org.tw

**Keywords:** sodium ion battery, cathode material, surpassing capacity, charge compensation, anionic redox process

## Abstract

A prismatic type layered Na_2/3_Ni_1/3_Mn_2/3_O_2_ cathode material for a sodium ion battery is prepared via two different methods viz., the solid state and sol–gel method with dissimilar surface morphology and a single phase crystal structure. It shows tremendous electrochemical chattels when studied as a cathode for a sodium-ion battery of an initial specific discharge capacity of 244 mAh g^−1^ with decent columbic efficiency of 98% up to 250 cycles, between the voltage range from 1.8 to 4.5 V (Na^+^/Na) at 0.1 C under room temperature. It is much higher than its theoretical value of 173 mAh g^−1^ and also than in the earlier reports (228 m Ah g^−1^). The full cell containing this material exhibits 800 mAh g^−1^ at 0.1 C and withstands until 1000 cycles with the discharge capacity of 164 mAh g^−1^. The surpassing capacity was expected by the anionic (oxygen) redox process, which elucidates the higher capacity based on the charge compensation phenomenon.

## 1. Introduction

Cost-effective storage of electrical energy is currently the global precedence in a range of extensive scientific and technological researches, wherein various rechargeable battery techniques have been developed in the past decades [1,2]. In particular, the Li-ion batteries have been considered as one of the most suitable candidates; however, the high cost of lithium and the geopolitical limit of lithium sources present tough challenges to LIBs to meet the worldwide demands. We are in a need of progressing towards the alternative energy storage devices, by the usage of abundant and environmental-friendly elements. Sodium-ion batteries have recently attracted much attention for electric energy storage, owing to its low cost and high abundance of sodium resources [3,4,5], also the Na-ion possesses similar electrochemical properties to the Li-ion. Therefore, sodium is a very attractive charge carrier to replace Li for the large scale energy storage applications, where the raw material cost becomes a dominant factor [6,7], and the cathode material is one of the key components, which plays a critical role in the performance of batteries. Earlier, various types of cathode materials have been prepared for the Na-ion, among them the layer structured cathode materials have engrossed much concern because of their high capacity with a sustainable cycle life equal to Li-ion.

The layered sodium metal oxides are classified by Delmas et al. [8] as P2- and O3-types, based on the occupying site of the Na-ion at prismatic and octahedral sites, respectively. The P2-type layered oxides such as Na_x_TMO_2_ and Na_0.7_CoO_2_ show higher initial capacity when compared to those of the O3-type NaMO_2_ (M = Co, Cr, etc.), but poor life cycle stability, huge capacity fading urged us towards the exertion on cathodes with improved electrochemical performances without toxic and costly metals such as Cr and Co, by replacing with safe, ecofriendly and high abundant materials like Mn and Fe. Besides, investigation of Lu and Dahn confirmed that the P2-layered oxide based on the Na-Ni-Mn-O system could provide a reversibly exchange and long cycle life with a high capacity (173 mAhg^−1^) for Na-ion cells [9,10,11,12,13,14].

The capacity of a cell is controlled by the redox potential of transition metal ions, but due to the charge compensation mechanism, the electron will be stored on the cation and anion, if the transition metal and the oxygen undergo the redox reactions, which allows the electrode to store higher capacity beyond the limits. The reports on the material Li_1.2_[Ni_0.13_^2+^Co_0.13_^3+^Mn_0.54_^4+^]O_2_ reveals that the redox reactions take place by transition metal ions and oxygen, which can store a capacity up to 230-250 mAhg^−1^. Recently, important studies on oxygen redox activity have been carried by Koga et al. [15] they have proposed that the oxygen loss at the surface due to the redox process is associated with densification and reversible oxygen redox activity that occurs on the materials without oxygen loss or a structural change of materials. Tarascon and co-workers [15,16,17,18,19,20] investigated these phenomena by substituting the 4d, 5d transition materials, which showed a higher charge storage capacity like the 3d series elements.

Moreover, Risthaus et al. [21] reported a high-capacity P2- Na_2/3_Ni_1/3_Mn_2/3_O_2_ cathode material for sodium ion batteries, which shows a discharge capacity of 228 mAh g^−1^, it is higher than the theoretical value 173 mAh g^−1^ of material. Herein, we have prepared the prismatic type layered Na2_/3_Ni_1/3_Mn_2/3_O_2_ cathode material by two different synthetic methods viz, solid state reaction (SSR) and sol–gel reaction (SGR), with improved surface morphologies, outstanding electrochemical performance as an excellent cathode material due to oxygen activity in the sodium ion battery [22]. Besides, this material delivers a surplus discharge capacity higher than the theoretical value and also previous reports from this effort, we have found that the larger surface area of the electrode material with reduced grain can lead to unique electrochemical performance with notable capacity [23]. It is also believed that a comparison and understanding with advantages and disadvantages of different preparation methods should lend a hand in designing efficient materials by suitable preparation route for promising electrode material to future development of a sodium ion battery.

## 2. Materials and Methods

### 2.1. Solid State Method (SSR)

The P2-type Na_2/3_Ni_1/3_Mn_2/3_O_2_ material was synthesized through a conventional solid-state reaction (SSR) using the stoichiometric amounts of Na (OOCCH_3_)_2_.4H_2_O (Alfa Aesar, 99.95%, heysham, England), Ni (OOCCH_3_)_2_.4H_2_O (Alfa Aesar 98+ %, heysham, England), and Mn (OOCCH_3_)_2_.4H_2_O (Alfa Aesar, 99%, heysham, England). The precursors were used as purchased deprived of supplementary cleansing. The mixtures ball milled well by RETSCH-PM-100 GmbH Planetary ball miller at 230 rpm for 4 h expending agate balls, latter for removing the acetate compounds, the resultant material was dried in vacuum oven at 120 °C for 2 h. Finally, powders were calcined at 950 °C at a heat rate of 3 °C/min in air for 12 h. Dark brownish color powder was obtained.

#### Sol–Gel Method (SGR)

The same sample was prepared by the sol–gel method (SGR) to prepare an aqueous solution, a stoichiometric amount of the three metal acetate salts (Mn, Ni, and Na; Alfa Aesar, >99.8%, heysham, England) were dissolved in distilled water, 1 M citric acid solution was then added drop wise as a chelating agent with vigorous stirring, and the pH level was maintained between 8 and 9 using ethylene diamine heated at 80 °C for 6 h. The obtained gel-like solution was kept in a vacuum oven at 120 °C for 6 h, and then the dried gel was calcined at 950 °C in a muffle furnace for 12 h under air. Finally, a dark black color sample was collected. In order to avoid contamination by moisture and humidity, the as-prepared powders were stored in an Argon (Ar) filled glove box.

### 2.2. Characterization Techniques

The crystal structure and phase purity of the prepared materials characterized by powder X-ray diffraction (XRD, (Malvern Panalytical, Leyweg, EA Almelo, The Netherlands)) using the PANalytical X’pertpro diffractometer with Cu Kα(1.54Å) radiation in the range 2θ = 10–80°. The crystal structure, lattice parameters and the unit cell volume of the synthesized material were obtained by Rietveld refinement using GSAS(GSAS/EXPGUI, USA) and structural visualization by VESTA software’s (ver. 3.4.4, Japan). Surface morphological characterization and elemental analyses were carried out by using a scanning electron microscope (EVO18 (CARL ZEISS) Jena, Germany) attached with the energy dispersive X-ray analysis (EDX Quantax 200 with X Flash^®^ 6130, elemental mapping). The grain size with a SAED pattern was analyzed via high resolution transmission electron microscopy (HR-TEM, JEOL, Musashino, Akishima-shi, Tokyo, Japan). The electronic state of different elements of prepared materials was analyzed by using ESCALAB 250xi with an XR6 Micro-focused Monochromator (Al Kα XPS, THERMO SCIENTIFIC, Waltham, Massachusetts, USA), XR4 Twin Anode Mg/Al (300/400 W) X-Ray Source, and using an EX06 Ion gun. All the electrochemical characterizations carried out by using a BCS-815/Electrochemical Analyzer (Bio-Logic, Claix, France).

### 2.3. Electrochemical Testing—Half-Cell Preparation

#### 2.3.1. Half-Cell Preparation

The electrodes for half-cells were prepared by slurry comprising of 85 wt % active material, 10 wt % conductive carbon black (Super P, Alfa Aesar 99.99%, heysham, England), and 5 wt % of binder (poly(vinylidene fluoride) (PVdF), Sigma-Aldrich, Saint Louis, USA) mixed with solvent N-methyl-2-pyrrolidone (NMP). The slurries prepared via both the methods were coated on aluminum (Al) foil by using an auto film coating machine with a heater setup (Model-MRX-TMH250, Shenzhen Ming Rui Xiang Automation Equipment Co., Ltd., Guangdong, China) that dried at 80 °C for 18 h. The mass loading of the active material in the electrodes was about 10 mg/cm^2^. The 2032 type half-cells were prepared with samples of P2-Na_2/3_Ni_1/3_Mn_2/3_O_2_ (both SGR, and SSR) as the cathode and the sodium metal as the anode. A 1 M solution of NaClO_4_ (sodium perchlorate, 98% Sigma-Aldrich, Saint Louis, USA) was dissolved in 1:1 wt % of EC/PC (ethylene carbonate/propylene carbonate, Alfa Aeser 99%, heysham, England) was used as an electrolyte and the high ionic conductive PVdF-SiO_2_ composite membrane was used as separator [24]. All the preparations were carried out in a glove box at inert atmosphere.

#### 2.3.2. Pouch Cell Preparation

The prototype pouch cell was prepared by using the SGR sample of P2-Na_2/3_Ni_1/3_Mn_2/3_O_2_ as a cathode and a tin-carbon (Sn/C) composite as an anode in the ratio of 2.5:1. The thickness, width, and length of the material loaded electrodes were 0.1, 40, and 150 mm, respectively. The electrodes were dried at 80 °C until the solvent was fully removed then it was coupled with a 90 µm thick PVdF-SiO_2_ composite separator and then folded. The folded electrodes placed into pouch with the dimension of 4 cm × 3 cm. Of the 1 M NaClO_4_ electrolyte 5 mL was injected into the pouch cell. The prepared pouch cell was kept at room temperature for 24 h under vacuum. In order to eliminate the surplus electrolyte and gas evolution in the pouch cell, it was punctuated under vacuum, and then it was sealed intact. Finally, the pouch cell was analyzed by electrochemical studies.

## 3. Results and Discussion

### 3.1. Structural Analysis

**Figure 1:**
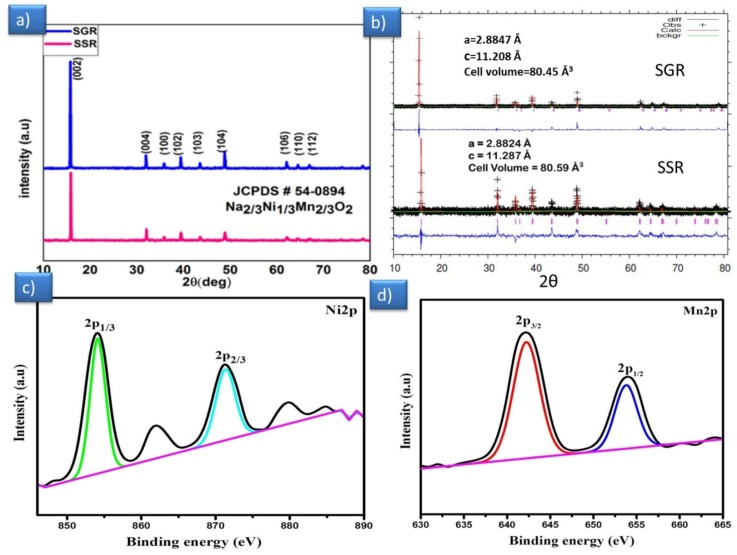
(**a**) X-ray diffraction of samples P2-Na_2/3_Ni_1/3_Mn_2/3_O_2_ (solid state reaction (SSR) and sol–gel reaction (SGR)), (**b**) refinement pattern with lattice parameters of P2-Na_2/3_Ni_1/3_Mn_2/3_O_2_ SGR, SSR respectively, and (**c,d**) XPS spectra of the P2-Na_2/3_Ni_1/3_Mn_2/3_O_2_ for Ni 2p and Mn 2p regions.

The crystalline structure was revealed by powder X-ray diffraction for the as-synthesized P2-type layered Na_2/3_Ni_1/3_Mn_2/3_O_2_ material samples (SSR and SGR), which is shown in Figure 1a. All the diffraction planes and sharp intense peaks correspond thriving to a hexagonal atomic lattice with the space group of P6_3_/mmc (No.194).

No secondary phases or impurities were detected and could be indexed to JCPDS No #54-0894. The stacking sequence of atom repetition was the ABBA (ABBA- ordering of metal oxides layer) metal oxide sheets. In the middle of the sheets, Na^+^ ions occupied two different sites by faces sharing (Na_f_) or edges (Na_e_) with the MO_6_ octahedrons of adjacent layers [23,25], which is demonstrating that pure and highly crystalline P2 type layered samples were synthesized by both the methods successfully. The unit cell volume and lattice parameters of the layered material obtained from refinement data by using Rietveld refinement listed out, which is in agreement with the literature [25,26] (Figure 1b). From the refined lattice parameter values, it is noticed that the layered P2-type structure was established by the MO_6_ (MO—metal oxides) octahedral layer and the trigonal prismatic configuration of the sodium cation coordinated with six oxygen atoms situated in between the transition metal layers. The electronic state of the different elements of prepared materials was analyzed by using XPS, and the analyzed XPS spectra of the materials P2-Na_2/3_Ni_1/3_Mn_2/3_O_2_ were shown in Figure 1c-d. The oxidation state of Ni and Mn on the surface of Na_2/3_Ni_1/3_Mn_2/3_O_2_ displayed in their regions separately. As shown in Figure 1c, the Ni 2p spectra display the Ni 2p_3/2_ binding energy lying in the region of 853.9 eV and for Ni 2p_1/2_, it lay in 871.2 eV, with their satellite peaks. The satellite peaks occurrence in the spectra of Ni 2p could be attributed to multi-electron excitation in line with the literature [27]. The Mn 2p peak was observed in the binding energy regions of 641.7 eV for Mn 2p_3/2_ (Figure 1d) and at 652.9 eV for the 2p_1/2_ orbital [28]. This elucidates that nickel and manganese had the 4+ with some 3+ electronic states.

### 3.2. Surface Morphological Analysis

**Figure 2:**
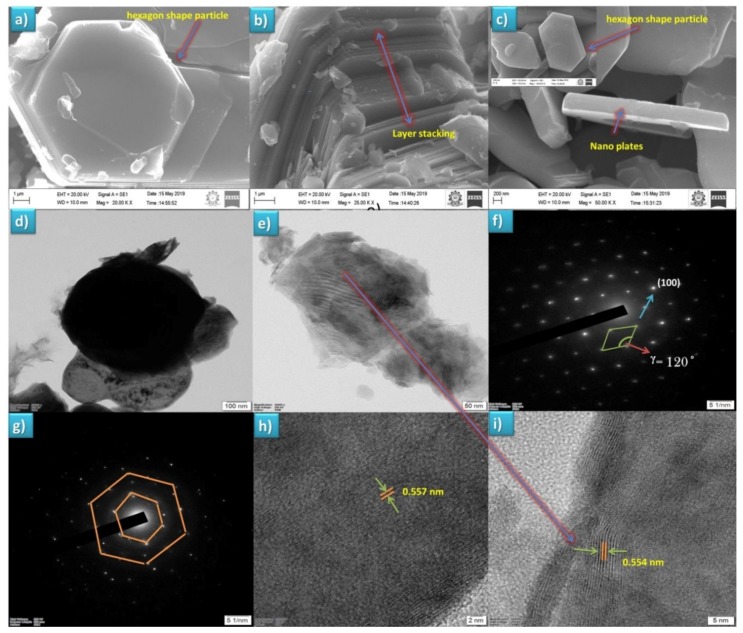
(**a**,**b**) SEM images of sample P2-Na_2/3_Ni_1/3_Mn_2/3_O_2_ prepared via the SSR method, (**c**) SEM images of sample P2-Na_2/3_Ni_1/3_Mn_2/3_O_2_ prepared via the SGR method, (**d**) HR-TEM image of sample P2-Na_2/3_Ni_1/3_Mn_2/3_O_2_ prepared via SSR, (**e**) HR-TEM image of sample P2-Na_2/3_Ni_1/3_Mn_2/3_O_2_ prepared via SGR, (**f**) SAED pattern of sample P2-Na_2/3_Ni_1/3_Mn_2/3_O_2_ prepared via the SGR method, (**g**) SAED pattern of sample P2-Na_2/3_Ni_1/3_Mn_2/3_O_2_ prepared via the SSR method, and (**h**,**i**) lattice fringes of samples P2-Na_2/3_Ni_1/3_Mn_2/3_O_2_ prepared via SSR and SGR, respectively.

A smooth surface morphology with a smaller grain size of the material is the prime key for achieving improved electrochemical performance for lithium/sodium-ion batteries [23]. The surface morphologies of the sample (SSR) were observed from secondary electron emission with the magnification of 25, and 50 k at a working distance 10.5 mm (Figure 2a–c); it was found with a hexagon shape with a layer sequence, which can accomplish the layer structure of the materials. In Figure 2a,c, the hexagon shaped particle surface had rough layer stacking and sparkle edges, which can be ascribed to the presence of sodium content charging due electron beam exposure [29]. The morphology of the sample SGR in Figure 2c depicts that the particle exhibited a nanoplate-like layer structure, which is in agreement with the report of Boisse et al. [30]. The energy dispersive X-ray analysis showed the presence of Na, Ni, Mn, and O elements in the as-synthesized layered Na_2/3_Ni_1/3_Mn_2/3_O_2_ material, which is provided in the supplementary information Furthermore, elemental mapping using SEM revealed the simultaneous occurrence of elements viz., Na, Ni, Mn, and O in the layered Na_2/3_Ni_1/3_Mn_2/3_O_2_ with appreciable distribution (supplementary information Figures S1 and S2). From this observation, the sample SGR prepared from the sol–gel method had a clean and smooth surface with a smaller particle.

#### HR-TEM Analysis

To elucidate the crystal structure and grain size, the as-prepared samples were analyzed by HR-TEM and shown in Figure 2d–i. Figure 2d,e reveals the average grain size was about 100 nm (SSR) and 50 nm (SGR). The difference in the grain size of the same samples indicates the effect of the preparation method, also suggesting the advantage of the solution based (SGR) preparation method for obtaining a reduced grain size in the nanoscale range. The lattice fringes were evidenced from Figure 2h,i with an interlayer distance of 0.557 nm (SSR) and 0.554 nm (SGR); this confirmed the high degree crystallization of the material from the SAED pattern of both samples. The hexagonal symmetry for the layered P2-Na_2/3_Ni_1/3_Mn_2/3_O_2_ in Figure 2f,g are typical diffraction patterns with the calculated gamma value (**ᵞ** = 120) for a hexagonal lattice of P2-Na_2/3_Ni_1/3_Mn_2/3_O_2_ [31].

### 3.3. Electrochemical Analysis

**Figure 3:**
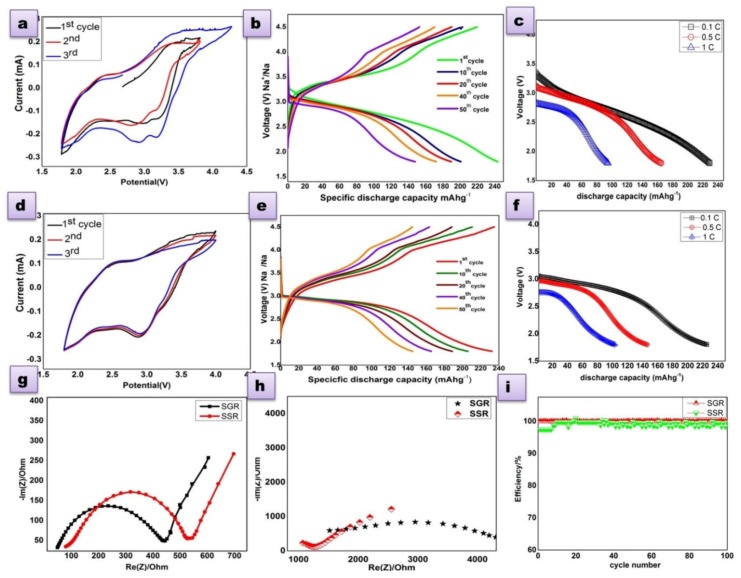
(**a**) Cyclic voltammetry of sample P2-Na_2/3_Ni_1/3_Mn_2/3_O_2_ prepared via the SSR method at a scan rate 0.1 mV/s, (**b**) charge discharge curve of sample P2-Na_2/3_Ni_1/3_Mn_2/3_O_2_ prepared via the SSR method, (**c**) rate capability of sample P2-Na_2/3_Ni_1/3_Mn_2/3_O_2_ prepared via the SSR method, (**d**) cyclic voltammetry of sample P2-Na_2/3_Ni_1/3_Mn_2/3_O_2_ prepared via the SGR method at the scan rate of 0.1 mV/s, (**e**) charge discharge curve of sample P2-Na_2/3_Ni_1/3_Mn_2/3_O_2_ prepared via the SGR method, (**f**) rate capability of sample P2-Na_2/3_Ni_1/3_Mn_2/3_O_2_ prepared via the SGR method, (**g**) electrochemical impedance spectrum (EIS) observed in the frequency range of 10 kHz to 10 Hz, (**h**) impedance of spectra after 250 cycles, and (**i**) columbic efficiency plot for the both sample of P2-Na_2/3_Ni_1/3_Mn_2/3_O_2_.

The electrochemical properties of P2 Na_2/3_Ni_1/3_Mn_2/3_O_2_ materials (SSR and SGR) were characterized with a 2032 coin type cell by various electrochemical techniques. The cyclic voltammetry profile shown in Figure 3a,d, were carried out between 1.8 and 4.5V at the scan rate of 0.1 mV/s. The redox peak in the potential range 3.0–3.5 V can be attributed to the redox reactions of Ni^2+^/Ni^3,4+^ with removable Na^+^ ions upon discharging less than 2.0 V, the peaks below 2.0 V may be due to the Mn redox process [32,33]. The charge/discharge properties carried out by the constant current cycling mode, in between 1.8 and 4.5 V vs. (Na^+^/Na) at a 0.1 C rate in room temperature are shown in Figure 3b,e. These materials show a surplus discharge capacity of 244 mAh g^−1^ with 98% efficiency, which is also higher than the theoretical value [21]. Moreover, the higher capacity rendition of this material confirmed by repeated observations. During the initial charging process, the higher capacity existence was due to the oxygen loss P2- Na_2/3_Ni_1/3_Mn_2/3_O_2_ framework by a charge compensation mechanism. Thus, the materials activated deliver a tremendous capacity throughout the subsequent discharge process; however, the length of the oxygen loss process may lead to a plateau at 3.8–4.2 V. The anionic redox process and charge compensation mechanism were discussed as follows. The rate capability test was carried at various C rates like 0.1, 0.5, and 1C as in Figure 2c,f. By fine observation, the sample SGR that had a lesser grain size shows more of a discharge capacity, this result designates that the reduced particles size sample SGR led to a good rate capability of the P2 Na_2/3_Ni_1/3_Mn_2/3_O_2_ material that purely depends on the particle size and large surface area of the material [34]. From this observation, the sol–gel method was an efficient preparation method, which can provide the materials in a much reduced size particle. The high columbic efficiency (CE) of the cell can be achieved by charge/discharge through moderate current rates; in this manner, here we achieved almost 98% columbic efficiency (CE) until 250 cycles at 0.1 C (Figure 3i). Further it is inferred from Figure 3i that the efficiency remained stable after changing the C rates, which could attribute to the stability of prepared P2- Na_2/3_Ni_1/3_Mn_2/3_O_2_ materials in both methods.

The kinetic property of sodium ions in the cell is revealed by electrochemical impedance spectroscopy. The Nyquist plots were taken before and after the charge/discharge of cell (Figure 3g,h); the impedance spectra of the cell entail a semicircle at the high frequencies region, which is attributed to charge transfer resistance (R_ct_), which is around 450 and 550 Ω for sample SGR and SSR respectively. The inclined line in the low frequency region indicates diffusion properties of the sodium ion through the cell [35]. The internal resistance of the cycled cell was huge in Figure 3h, due to the solid electrolyte interface layer formation on the electrode surface, which is formed during the shuttling process of ions [36].

#### 3.3.1. Full-Cell

**Figure 4:**
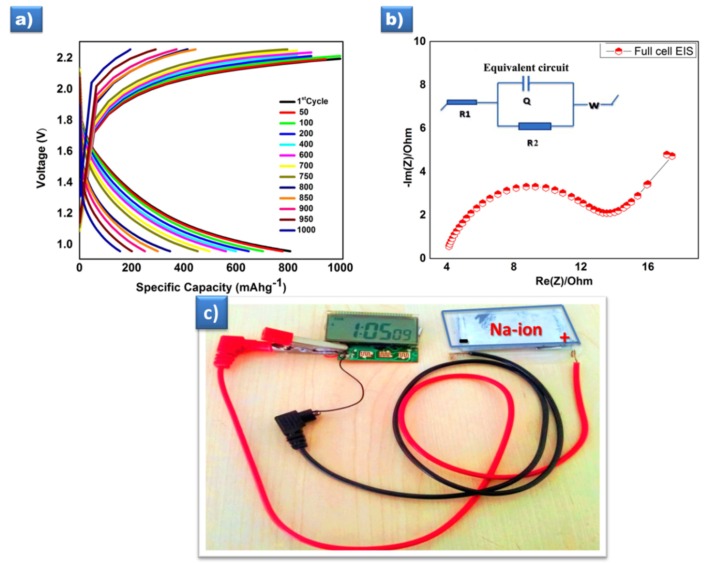
(**a**) Charge discharge curve of a prototype full-cell cycled between 1.0-2.2V at 0.1 C, (**b**) Electrochemical impedance Spectra of the full-cell setup with an equivalent circuit, and (**c**) the prototype of Na-ion full-cell powering the digital device.

As described in the electrochemistry section, the sample SGR tested with a prototype full cell with the Sn/C anode delivered a higher capacity around 800 mAh g^−1^ at the initial cycle (Figure 4a), when cycled between 1.0 and 2.2 V at 0.1 C and sustained at 164 mAh g^−1^ even after the 1000th cycle. It is higher than the earlier report of 650 mAh g^−1^ for this material [37]. The impedance spectrum and the equivalent circuit of the full cell shown in Figure 4b indicate the high ionic conducting nature of the battery with a lower charge transfer resistance R_ct_.

From all the above discussions, this abundant capacity of P2- Na_2/3_Ni_1/3_Mn_2/3_O_2_ materials have been reported for the first time to the best of our knowledge for both the half and full cell, which is very clear from the comparison (Table 1). Risthaus et al. [21] reported that a high capacity P2 Na_2/3_Ni_1/3_Mn_2/3_O_2_ cathode material delivers a discharge capacity of 228 mAh g^−1^ within 1.5–4.5 V at a 0.05 C rate in half cells, which is higher than the theoretical value due to only the redox reaction of Ni^2+^/Ni^4+^, also either Mn or oxygen redox activity. Moreover, the prepared prototype has a working voltage of 2.2 V, and specific energy and power density of 207.6 W h kg^−1^ and 1650 W kg^−1^, respectively. Most impressively, the appreciable cycling stability reached up to 1000 cycles at the 0.1 C current rate with negligible capacity loss.

#### 3.3.2. Charge compensation mechanism

Hy et al. [38] established the higher capacity due to the charge compensation mechanism by the oxygen redox process in the Li rich layer oxide materials. Earlier, Bruce et al. [39] and Ceder et al. [40] made a mark on this material P2-Na_2/3_Ni_1/3_Mn_2/3_O_2_ and reasoning that the high capacity was due to the participation of oxygen in the redox reaction by also the charge compensation mechanism.

**Figure 5:**
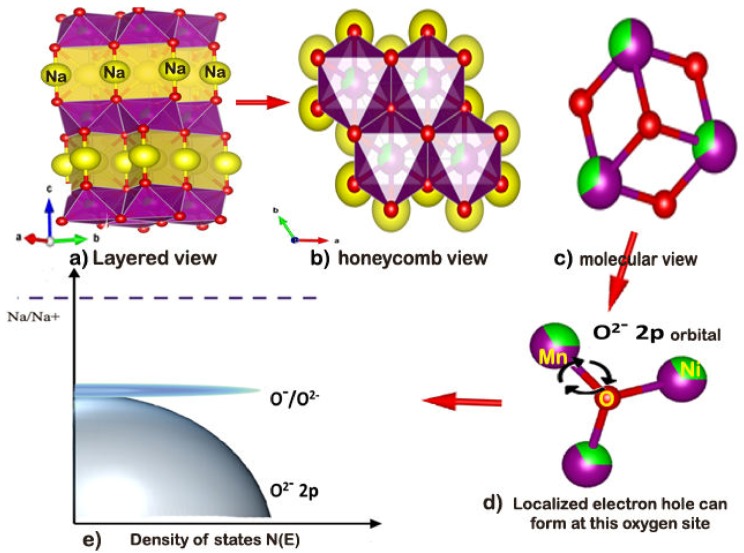
Schematic of the charge compensation mechanism by anionic redox. (**a**) Layered structure of P2-Na_2/3_Ni_1/3_Mn_2/3_O_2_ where the sodium ion bonded between metal oxide slabs. (**b**) Honeycomb view of the cation ordering in the metal oxide layer in P2-Na_2/3_Ni_1/3_Mn_2/3_O_2_ viewed along the c-axis. (**c**,**d**) molecular view of oxygen coordination by Mn^4+^ and Ni^2+^ and these promote a localized electron hole on oxygen due to a relative ionic Mn^4+^/–O interaction. (**e**) Schematic of energy versus the density of states, which can show the position of O^−^/O^2−^.

The charge compensation can be understood from the above pictographic representation as shown in Figure 5a–e, the localized electron-hole form on the oxygen atom of material. In Figure 5d, the oxygen surrounded by the metal ions like Mn/Ni/Na atoms by an ionic bond where they were localized near to the top of the valence bond O^2−^ as in Figure 5e. In the Na rich transition materials, localized electron holes can be formed on the oxygen atoms by extraction of Na^+^ ions from metal oxide frame of cathode upon charging above 3.9 V (phase evaluation cut off voltage P2-O2). The basic requirement of the anionic redox process is that the unhybridized 2p orbital can present in oxygen the strong ionic bonding state of Na–O–Na. This reveals that the electronegativity difference of Na–O should be above 2.0. In the P2-type materials, there was a larger interlayer repulsion force accrued due to the same layer (ABBA—Figure 1c) stacking of the TM (TM-Transition metal) layers, which also led to free kinetics to larger ionic Na^+^ ions during the intercalation. According to Koga et al. [15], the oxygen losses at the surface due to the redox process was confirmed by calculating the oxygen atomic percentage of the electrode surface, before and after the cycling process, by using the X-ray energy dispersive analysis performed on various areas on the as-prepared electrode. It is noted that the average of the oxygen atom for the fresh and cycled electrode was 50.4% and 35.85% respectively (Figures S3 and S4 and Table S1). The estimated difference of 14.55% was due to the participation of the oxygen atom in the redox process during the charge/discharge of the cell. The charge compensation mechanism will guide the design of new transition metal oxide cathodes, significantly higher capacity for future sodium ion batteries.

## 4. Conclusions

In this work, we prepared layered P2-Na_2/3_Ni_1/3_Mn_2/3_O_2_ materials by two different methods with different surface morphologies, which show a surplus capacity with good columbic efficiency. The excellent cycling property and high rate capability were observed for the sample prepared via a sol–gel reaction (SGR) when compared to the sample prepared through a solid state reaction (SSR). To the best of our knowledge, this work is the first study that investigates the maximum capacity (244 mAhg^−1^ (half-cell) and 800 mAhg^−1^ (full-cell)) for this material. This excellent electrochemical performance of this material was due to the anionic redox reaction and the charge compensation mechanism. Oxygen was conventionally inactive; however, the existence of oxygen in the redox process in this layered P2-Na_2/3_Ni_1/3_Mn_2/3_O_2_ material could actively contribute to the enormous energy storage. This P2-Na_2/3_Ni_1/3_Mn_2/3_O_2_ material could be served as a paramount alternative for high cost Li-ion batteries, with low cost, high abundance, and excellent electrochemical performances.

## Figures and Tables

**Table 1:** Comparison of capacities of P2-Na electrodes reported in the literature for half-cell and full-cell couple.

Half-Cell Comparison									
S. No.	Cathode Materials		Capacity (mAhg^−1^)	Voltage (V)		Electrolyte Used			Ref.
1	P2-Na_0.67_Mn_0.65_Fe_0.2_Ni_0.15_O_2_		208	2.0–4.0		1.0 M NaPF_6_ (EC/DEC, 50: 50 vol%)			[41]
2	P2-Na_2/3_ [Ni_1/3_Mn_2/3_] O_2_		134	2.0–4.0		1.0 M NaClO_4_ (PC) + (FEC) (95:5, v/v)			[42]
3	P2-Na_0.67_Mn_0.65_Ni_0.1_Co_0.15_O_2_		155	1.5–4.2		1.0 M NaClO_4_ in propylene carbonate (PC)			[43]
4	P2- Na_0.66_Ni_0.33-x_Zn_x_Mn_0.67_O_2_		132	2.0–4.2		1 M NaClO_4_ dissolved in PC with 2 vol.% FEC			[44]
5	P2- Na_2/3_Mn_1-x_Al_x_O_2_		162	2.0–4.0		1 mol NaClO_4_ in EC and PC was 1:1 in volume			[45]
6	P2-Na_0.4_Mn_0.54_Co_0.46_O_2_		194	1.5–4.0		1 mol NaClO_4_ in (EC/DEC = 4:6 in volume)			[46]
7	P2- Na_2/3_Ni_1/3_Mn_2/3_O_2_		228	1.5–4.5		1M NaPF_6_ in 1/1 weight ratio (EC/DMC)			[21]
8	P2- Na_2/3_Ni_1/3_Mn_2/3_O_2_		244	1.8–4.5		1M NaClO_4_ in 1/1 weight ratio in EC/PC			This Work
**Full-Cell Comparison**									
**S. No.**	**Cathode**	**Anode**			**Capacity (mAhg^−1^)**		**Voltage (V)**	**Type of Cell**	**Ref.**
**1**	Na_0.83_Ni_0.40_Ti_0.60_O_2_	Na_0.83_Ni_0.40_Ti_0.60_O_2_			100		0.01–2.0	Coin-2032	[47]
**2**	Na_0.7_CoO_2_	Graphite			80		0.5–3.7	Coin-2032	[48]
**3**	NaNi_1/2_Mn_1/2_O_2_	Hard carbon			250		0.7–2.0	Coin-2032	[49]
**4**	Na_0.76_Ni_0.3_Fe_0.4_Mn_0.3_O_2_	Hard carbon			650		1.5–3.8	Pouch cell	[37]
**5**	P2-Na_2/3_Ni_1/3_Mn_2/3_O_2_	Tin-Carbon			750		1.0–2.2	Pouch cell	This work

## Supplementary Materials

The following are available online at https://www.mdpi.com/2073-4360/12/3/662/s1, Figure S1-shows the occurrence of elements of sample SSR sodium (Na), nickel (Ni), manganese (Mn), oxygen (O) respectively, and the EDX shows the presence of respective elements. Figure S2-shows the occurrence of elements of sample SGR sodium (Na), nickel (Ni), manganese (Mn), oxygen (O) respectively, and the EDX shows the presence of respective elements. Figure S3. (a,b) SEM images of electrode before charge discharge, (c,d) after charge discharge at scale of 20 µm. Figure S4. (a,b) EDX- image of pristine and cycled electrode and (c, d) EDX spectra with presence of elements after charge discharge. Table S1. Summary of electrode elements before and after cycling and atomic loss of sodium (Na), oxygen (O) in electrode through EDAX.
