# Supplementary material for: High Capacity Prismatic Type Layered Electrode with Anionic Redox Activity as an Efficient Cathode Material and PVdF/SiO2 Composite Membrane for a Sodium Ion Battery
Source: Polymers (Basel). 2020 Mar 16;12(3):662. doi: 10.3390/polym12030662 (PMC7183058; doi:10.3390/polym12030662)
Supplement: Supplementary file 1 [file polymers-12-00662-s001.pdf]

Supplementary Information

High Capacity Prismatic Type Layered Electrode with Anionic Redox Activity as an Efficient Cathode Material and PVdF/SiO_2 Composite Membrane for a Sodium Ion Battery

Arjunan Ponnaiah[a], Subadevi Rengapillai [a,*], Diwakar Karuppiah [a], Sivakumar Marimuthu [a,*], Wei-Ren Liu [b] and Chia-Hung Huang [c]

[a] #120, Energy Materials Lab, Department of Physics, Science Block, Alagappa University, Karaikudi-630 003, Tamil Nadu, India; nano.arjun@gmail.com (A.P.); selfindicator@gmail.com (D.K.)

[b] Department of Chemical Engineering, R&D Center for Membrane Technology, Research Center for Circular Economy, Chung-Yuan Christian University, Chung-Li-32023, Taiwan, ROC; wrliu@cycu.edu.tw

[c] Metal Industries Research and Development Centre, Kaohsiung-81160, Taiwan; chiahung@mail.mirdc.org.tw

* Correspondence: susiva73@yahoo.co.in (S.M.); susimsk@yahoo.co.in (S.R.)

1. Fig.S1- EDX spectra and Element Overlay-Mapping of the sample prepared via solid state reaction

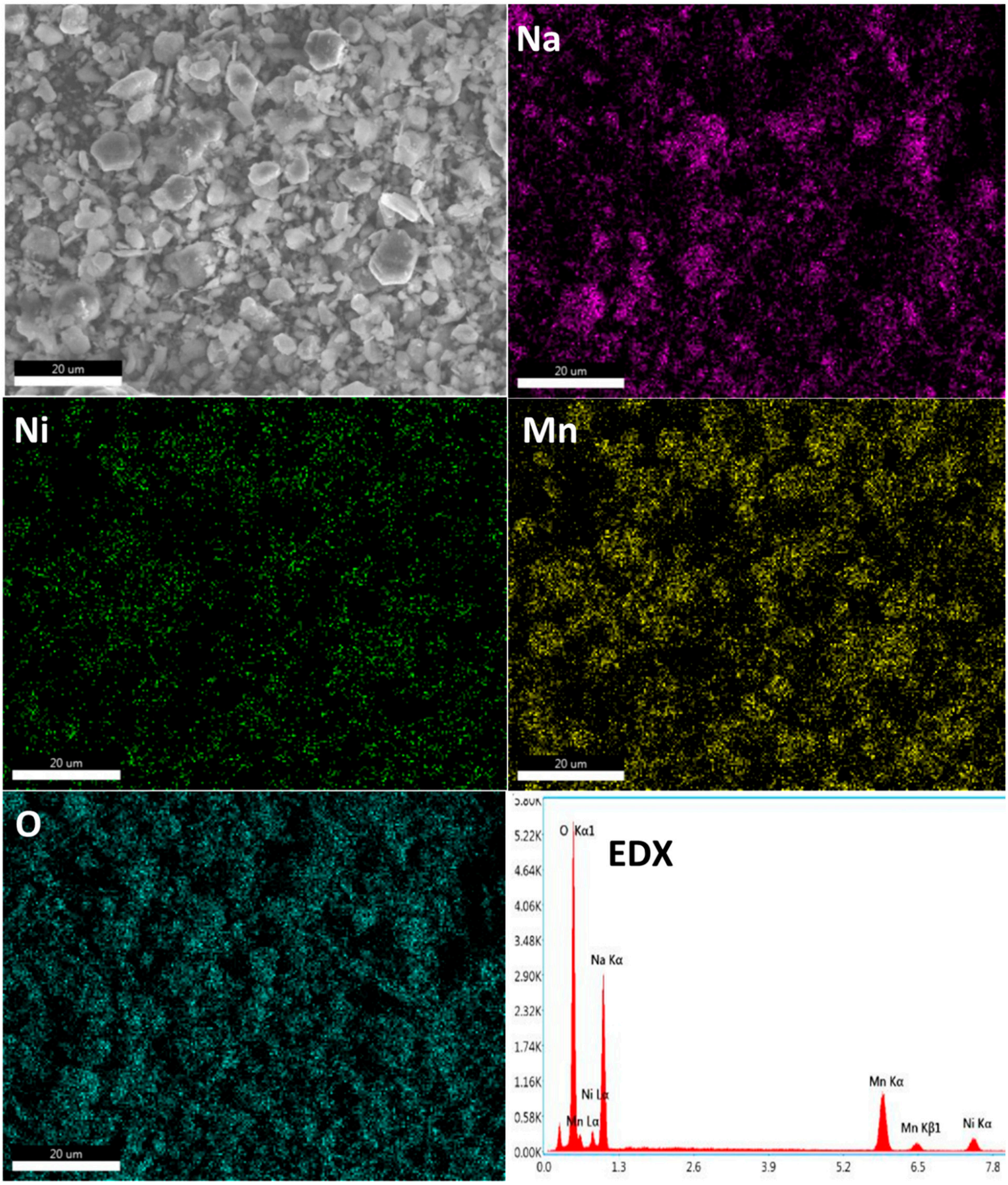

Fig.S1-shows the occurrence of elements of sample SSR sodium (Na), nickel (Ni), manganese (Mn), oxygen (O) respectively, and the EDX shows the presence of respective elements.

2. Fig.S2- EDX spectra and Element Overlay-Mapping of the sample prepared via solid state reaction

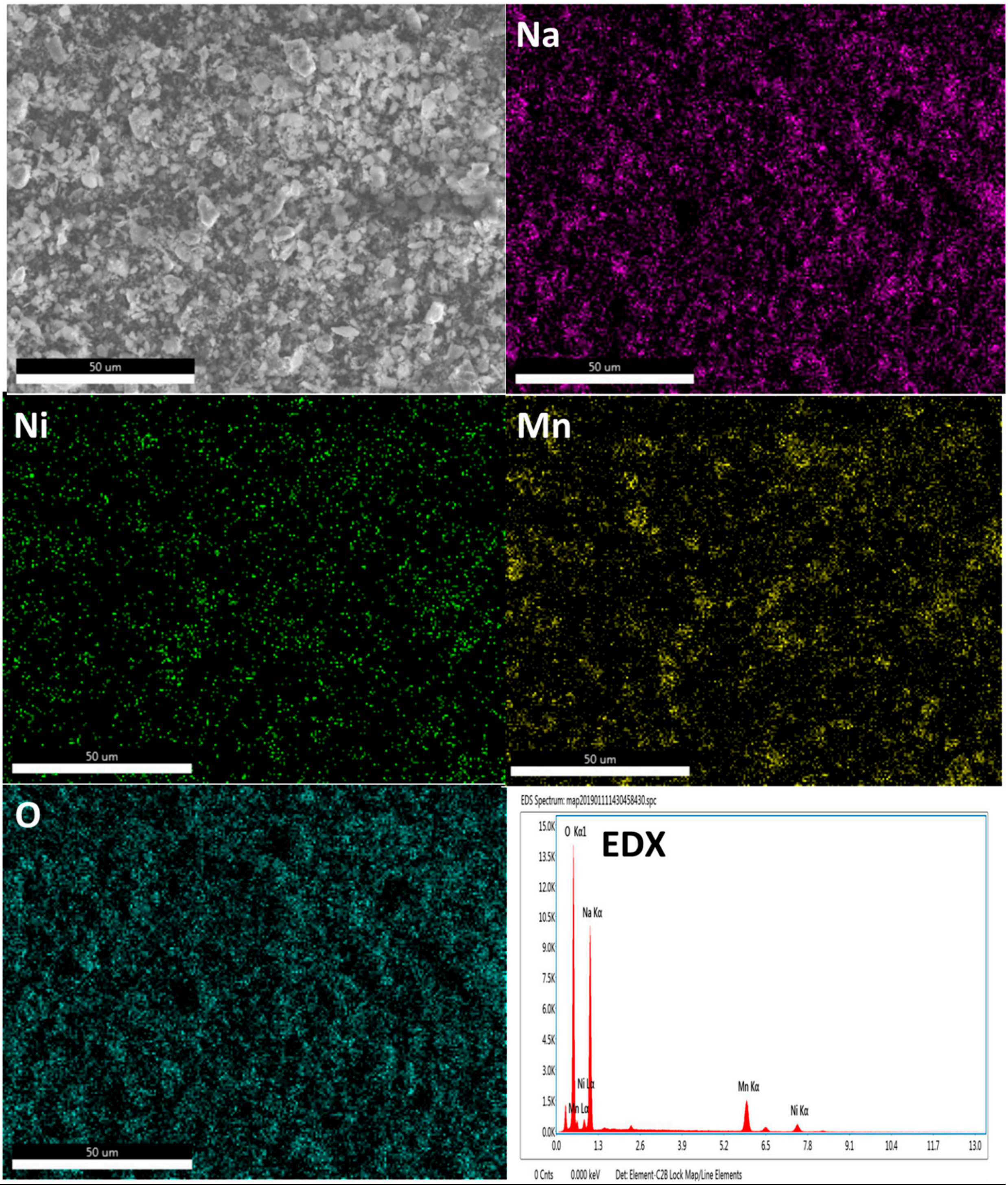

Fig.S2-shows the occurrence of elements of sample SGR sodium (Na), nickel (Ni), manganese (Mn), oxygen (O) respectively, and the EDX shows the presence of respective elements.

3. Fig.S3. Scanning electron microscopic (SEM) images of prepared electrode

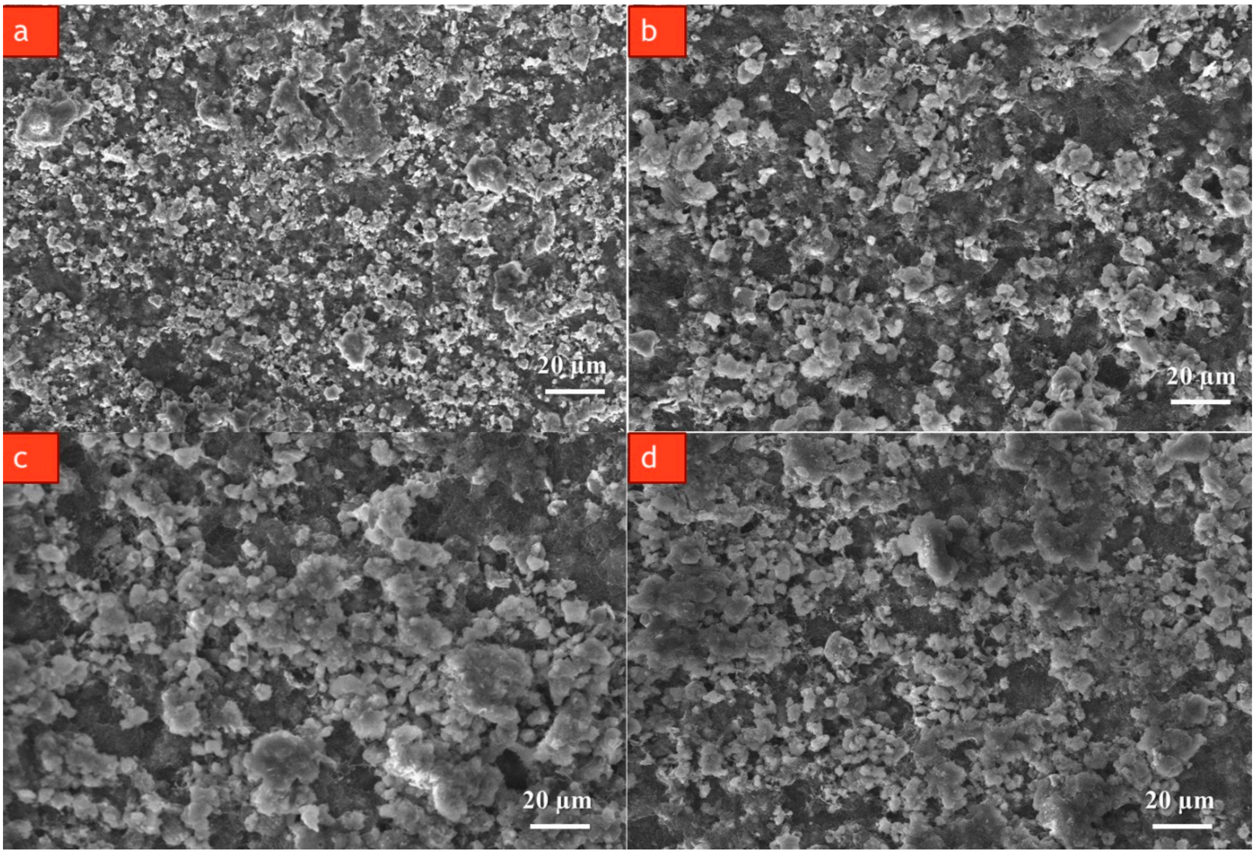

Fig.S3. (a-b) SEM images of electrode before charge discharge, (c-d) after charge discharge at scale of 20 μm

4. Fig.S4. X-ray energy dispersive micro elemental analysis of electrode after charge discharge.

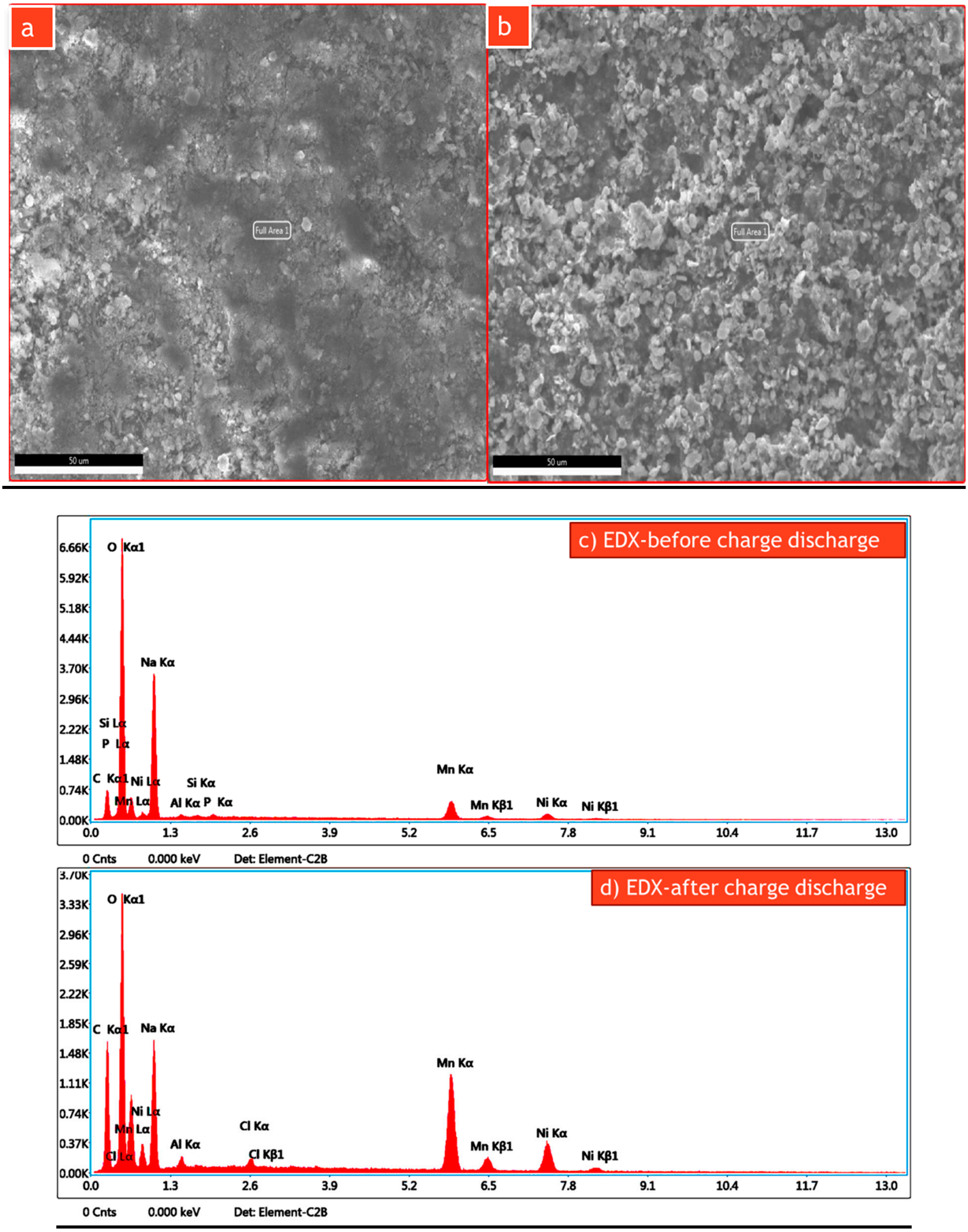

Fig.S4. (a, b) EDX- image of pristine and cycled electrode and (c, d) EDX spectra with presence of elements after charge discharge

5. Table.T1. Summary of electrode elements before and after cycling and atomic loss of sodium (Na), oxygen (O) in electrode through EDAX.

Atomic %(Scanned Area of Electrode)-EDX Data													
**Element**	**Pristine Electrode**						**Cycled Electrode**						**Atomic Loss In %**
	**Area 1**	**Area 1a**	**Area 2**	**Area 2a**	**Total**	**Average**	**Area 1**	**Area 1a**	**Area 2**	**Area 2a**	**Total**	**Average**	
**Oxygen**	49.6	53.1	46.6	52.3	201.6	50.4	31.2	39.8	33.2	39.2	143.4	35.85	14.55
**Sodium**	26.0	41.0	25.7	39.7	132.4	33.1	14.5	39.5	14.6	38.2	106.8	26.7	6.4
